# Prognostic significance of TMEM16A, PPFIA1, and FADD expression in invasive ductal carcinoma of the breast

**DOI:** 10.1186/1477-7819-12-137

**Published:** 2014-05-01

**Authors:** Eun Ji Choi, Jeong A Yun, Sahrish Jabeen, Eun Kyoung Jeon, Hye Sung Won, Yoon Ho Ko, Su Young Kim

**Affiliations:** 1 Department of Pathology, The Catholic University of Korea, School of Medicine, Seochogu Banpodaero 222, Seoul 137-701, South Korea; 2 Department of Internal Medicine, The Catholic University of Korea, School of Medicine, Seoul, South Korea

**Keywords:** TMEM16A protein, PPFIA1 protein, FADD protein, Carcinoma, Ductal, Breast

## Abstract

**Background:**

11q13 region is a frequently amplified locus in human malignancies. Among the genes located in this region, *FADD* is one of the alleged driving genes. Because amplification is not generally confined to a single gene and amplified genes may not show increased expression, we need to evaluate clinical significance of changes occurring in 11q13 region to understand their roles in carcinogenesis. Therefore, we screened expressions of *FADD* and closely located genes (*PPFIA1* and *TMEM16A*) and evaluated the expressions to find clinical significance in invasive ductal carcinoma of the breast.

**Methods:**

Ninety-eight cases of invasive ductal carcinoma of the breast were collected. Using archival tissues resected from the cases, we built a tissue microarray and used it in immunohistochemistry. We evaluated the association of *FADD*, *PPFIA1*, and *TMEM16A* expression scores with clinicopathological parameters, including disease-free survival.

**Results:**

*FADD* expression was associated with T stage (*P* = 0.046). The combined score of *FADD*, *PPFIA1*, and *TMEM16A* gene expressions was associated with perineural invasion (*P* = 0.022). Although individual gene expressions of TMEM16A, FADD, and PPFIA1 failed to show significant association with disease-free survival, combined gene expression scores did show association with disease-free survival (*P* = 0.034).

**Conclusions:**

*FADD*, *TMEM16A*, and *PPFIA1* gene expressions as a whole were associated with disease-free survival in breast cancer.

## Background

Amplification is one of the activating mechanisms of proto-oncogenes during carcinogenesis [1]. Among the frequently amplified regions in human malignancies, amplifications of 11q13 were found in breast cancers [2-4], esophageal squamous cell carcinoma [5], and head and neck cancers [6-9]. In this region, several genes were located and the amplified region encompassed more than one of the tested loci [3]. In addition, region q13 of chromosome 11 was pointed out to have a high penetrance gene for breast cancer in the study of 19 non-*BRCA1/2* families [10]. However, amplification does not necessarily result in increased expression of the affected gene. Taking into account that gene products rather than the genes themselves are effector molecules controlling biological behavior of the cells, we need to know the expression changes of the genes located in 11q13 and their clinical significance to understand their contribution to tumor cell behavior.

Among the genes located in the region of 11q13, *FADD* was reported as a driver in the 11q13 amplicon in laryngeal/pharyngeal cancer [11]. FADD is an adaptor molecule interacting with many kinds of death receptors and induces apoptosis by caspase-8 [12,13]. FADD expression was associated with metastasis in squamous cell carcinoma of the head and neck [14] and poor prognosis in oral squamous cell carcinoma [15] and lung adenocarcinoma [16]. Because the amplification of *FADD* does not confined in a single gene, *TMEM16A* or *PPFIA1*, which are in close proximity to *FADD*, may be amplified concomitantly. In addition, the BAC clone (RP11-203 N8), which is used as a template when synthesizing the fluorescence *in situ* hybridization (FISH) probe for *FADD*, includes the regions of *TMEM16A* and *PPFIA1*. Confirmation of amplification by FISH cannot differentiate copy number alterations among the genes. The locus encompassing these three genes is amplified in several malignancies, including breast cancer [17].

*TMEM16A* is associated with activation of the calcium dependent chloride channel and regulates cell proliferation [18,19]. *TMEM16A* is considered to be proto-oncogenic and increases tumor growth when overexpressed in gastrointestinal stromal tumors [20], and head and neck cancer [18]. Previous reports claimed that the calcium chloride channel influenced the growth of tumors and loss of *TMEM16A*, leading to decrease in tumor size [18,21]. However, the mechanism of tumor growth by *TMEM16A* overexpression is not known [22].

PPFIA1 is a member of the LAR protein-tyrosine phosphatase-interacting protein family. PPFIA1 is amplified in human breast cancers and promotes invasiveness of breast and cervical cancer cells [23]. Also, PPFIA1 acts as a tumor suppressor and regulates cell motility by interacting with ING4 [24]. When expression of PPFIA1 was reduced, invasiveness of head and neck squamous cell carcinomas (HNSCC) was found to increase [25].

Although *TMEM16A*, *FADD* and *PPFIA1* are co-amplified in many malignancies, the combined effect of the three gene expressions has not been evaluated to date. To understand the net effect of these three gene alterations, we screened the gene expressions by immunohistochemistry and analyzed the association with clinicopathological parameters, including disease-free survival.

## Methods

### Patients and tumor tissues

We collected 98 cases of invasive ductal carcinoma surgically resected at Uijeongbu Mary’s Hospital from 2002 to 2004. Patients’ age was between 29 and 77 (mean, 49.1) years old. Forty-six cases were treated with adjuvant chemotherapy and 33 cases received hormone therapy. Adjuvant radiotherapy was given to 22 cases. Disease-free survival data (11.0 to 103.3 months; mean, 61.9 months) was available. The disease recurred in 21 cases and 7 cases died of the disease. We selected representative archival tissues resected from the cases and tissue microarray was constructed using manual tissue arrayer, MTA-1 (Estigen Tissue Science, Estonia). Human tissue acquisition and its use followed the Institutional Review Board-approved protocol (CUMC11U058) at the Catholic University of Korea, School of Medicine.

### Immunohistochemistry

The immunohistochemical staining of breast cancer tissue followed the previously reported protocol [26]. Briefly, tissue sections were cut in 4-μm thicknesses and transferred to ProbeOn Plus slides (Fisher Scientific, Pittsburgh, PA, USA). To minimize tissue loss during boiling procedure and to get rid of excess paraffin on the slides, tissues were incubated for two hours in a 56°C dry chamber (Agilent Technologies, Santa Clara, CA, USA). The sections were deparaffinized in xylene three times and hydrated through 100%, 90%, 80%, 70% ethanol and Tris-buffered saline (TBS, pH 7.4). To make the epitopes more accessible to the primary antibodies used in the current study, the tissues were boiled in 10 mM sodium citrate buffer (pH 6.0) using a microwave for 20 minutes. To quench endogenous peroxidase, we treated the tissues with 3% hydrogen peroxide in PBS. The tissues were incubated with the respective primary antibodies at 4°C overnight (Table 1). After incubating the tissue with biotinylated secondary antibody, diluted (1:50) ExtrAvidin (Sigma-Aldrich, St. Louis, MO, USA) was used to amplify signal intensity. For visualization, liquid DAB + substrate chromogen system (Dako, Glostrup, Denmark) was used. Scoring of immunohistochemical staining was divided into three groups. Positive staining in less than 5% of tumor cells was considered negative. Cases showing a brown color in more than 50% of tumor cells were considered to be a strongly positive group. Cases showing light brown color in more than 5% and dark brown color in less than 50% of tumor cells were counted as a weakly positive group [14,20].

**Table 1:** Primary antibodies used in immunohistochemistry

**Target**	**Dilution ratio**	**Host**	**Clone**	**Provider**
PPFIA1	1:100	Rabbit		Proteintech (Chicago, IL, USA)
TMEM16A	1:1	Rabbit	SP31	Abcam (Cambridge, UK)
FADD	1:10	Rabbit	EP887Y	Abcam

### Statistical analysis

Where appropriate, the Chi-square test or Fisher’s exact test was used to evaluate association of immunoreactivity with clinicopathologic parameters. For disease-free survival analysis, Kaplan-Meier method and the nonparametric log-rank test was used. We used R ver. 3.0.2 (R foundation, Vienna, Austria) for statistical tests and their graphic presentations.

## Results

### Patient characteristics

Among 98 cases in total, 5 (5.1%) cases of grade I, 51 (52.0%) cases of grade II, and 40 (40.8%) cases of grade III were included in this study. Two (2.0%) cases did not have information on grade status. T1, T2, and T3 stages were 23 (23.5%), 64 (65.3%), and 11 (11.2%) cases, respectively. For nodal stages, N0 (41 cases, 41.8%) and N1 (34 cases, 34.7%) were most common. Estrogen receptor (ER) was positive in 63 cases and progesterone receptor (PR) was positive in 65 cases (Table 2). ER and PR positivities of nine cases were not known.

**Table 2:** The pathological parameters of the patients

**Pathological parameters**	**Number of cases (total 98 cases)**	
Age		
< 50	59	
≥ 50	39	
Grade		
I	5	
II	51	
III	40	
T stage		
T1	23	
T2	64	
T3	11	
N stage		
N0	41	
N1	34	
N2	17	
N3	6	
Estrogen receptor (ER)		
Negative	26	
Positive	63	
NA^a^	9	
Progesterone receptor (PR)		
Negative	24	
Positive	65	
NA^a^	9	
PNI		
Positive	19	
Negative	71	
NA^a^	8	
LVI		
Positive	65	
Negative	29	
NA^a^	4	

^a^NA: not available. LVI, lymphatic vessel invasion; PNI, perineural invasion.

### TMEM16A, FADD, and PPFIA1 immunoreactivity

**Figure 1:**
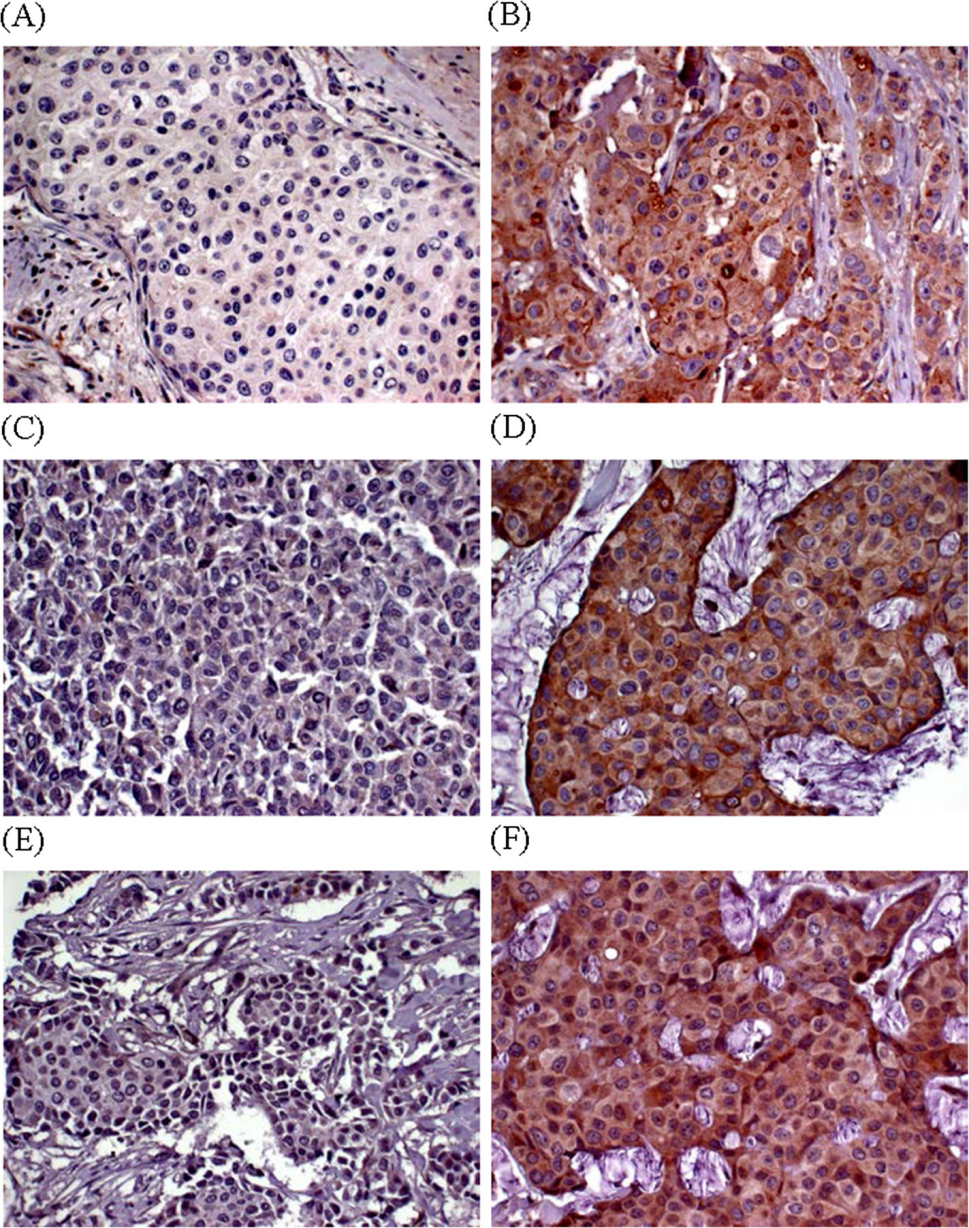
**Representative FADD, TMEM16A, and PPFA1 expression in breast cancer. (A)** FADD, negative. **(B)** FADD, positive. **(C)** PPFIA1, negative. **(D)** PPFIA1, positive. **(E)** TMEM16A, negative. **(F)** TMEM16A, positive.

TMEM was positive in 86 cases (strongly positive 5 cases, weakly positive 81 cases). FADD was positive in 62 cases (strongly positive 3 cases, weakly positive 59 cases). PPFIA1 was positive in 88 cases (strongly positive 24 cases, weakly positive 64 cases) (Figure 1). Strongly positive and weakly positive groups were pooled in the positive group for association tests and survival analysis. In association analysis of immunoreactivity of each protein and pathological parameter, significant association was only found between FADD expression and T stage (*P* = 0.046). Neither TMEM16A nor PPFIA1 showed significant association with any pathological parameters studied (Table 3). To see if the expressions of the three genes are associated, we calculated correlation of coefficient of each expression. TMEM16A and PPFIA1 expression was marginally correlated (r = 0.6). However, FADD expression showed low correlation with PPFIA1 and TMEM16A expression (r = 0.35, each).

**Table 3:** TMEM16A, FADD, and PPFIA1 expressions in invasive ductal carcinoma in relation to clinicopathological parameters (n = number of cases)

	**TMEM16A**			**FADD**			**PPFIA1**			**Hazard score**^**c**^		
	**+**	**-**	***P***	**+**	**-**	***P***	**+**	**-**	***P***	**< 2**	**≥ 2**	***P***
T stage			0.284			0.046^d^			0.444			0.069
1	22	1		19	4		22	1		20	3	
2 to 3	64	11		43	32		66	9		50	25	
N stage			0.756			0.277			1			0.316
0	37	4		29	12		37	4		32	9	
1 to 3	49	8		33	24		51	6		38	19	
Grade			0.756			0.285			1			0.197
1 to 2	48	8		38	18		50	6		43	13	
3	36	4		22	18		36	4		25	15	
ER			0.724			0.576			0.439			1.000
0	22	4		15	11		22	4		19	7	
1	56	7		42	21		58	5		45	18	
PR			0.266			1.000			0.244			0.898
0	19	5		15	9		20	4		18	6	
1 to 3	59	6		42	23		60	5		46	19	
HER2			1.000			0.918			1.000			0.831
0	46	7		33	20		47	6		37	16	
1 to 3	31	4		23	12		32	3		26	9	
Age			0.758			1.000			0.723			0.453
< 50	51	8		37	22		54	5		40	19	
≥ 50	35	4		25	14		34	5		30	9	
LVI^a^			1.000			0.746			0.716			0.564
0	26	3		17	12		27	2		19	10	
1 to 2	58	7		42	23		58	7		48	17	
PNI^b^			0.682			0.077			0.678			0.022^a^
0	62	9		48	23		63	8		16	55	
1	18	1		8	11		18	1		10	9	

^a^Lymphatic vessel invasion.

^b^Perineural invasion.

^c^Hazard score = TMEM16A score + PPFIA1 score – FADD score.

^d^*P* < 0.05.

ER, estrogen receptor; HER2, human epidermal growth factor; PR, progesterone receptor.

### Disease-free survival analysis

Survival difference was significant in grade (*P* = 0.01) and N stage (*P* = 0.00) but not in age (*P* = 0.85) and T stage (*P* = 0.189). Both PPFIA1 and TMEM16A expression showed tendency of association with poor survival group (*P* = 0.161 and 0.114, respectively). However, the survival differences were not strong enough to show statistical significance. For FADD expression, positive expression showed a tendency towards better survival, but this also failed to show statistical significance (*P* = 0.182).

### Combined effect of TMEM16A, PPFIA1, and FADD expressions

To evaluate net effect of the closely located genes, the score representing combined effect of the three gene expressions was calculated and we named it ‘hazard score’. Because FADD expression showed a tendency towards better patient survival, we calculated a combined score using the following equation:

hazardscore=TMEM16Ascore+PPFIA1score-FADDscore

**Figure 2:**
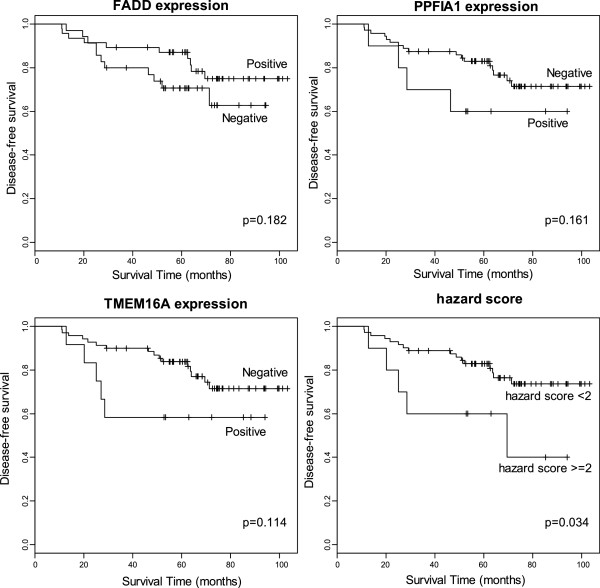
Disease-free survivals between groups with different expressions of FADD, PPFIA1, TMEM16A, and hazard score*. *Hazard score = TMEM16A score + PPFIA1 score – FADD score.

We found the hazard score was associated with perineural invasion status of the cases (Table 3). The cases with the hazard scores of 2 or more showed significant association with poor disease-free survival (*P* = 0.034, Figure 2).

## Discussion

In the current study, we screened the expressions of TMEM16A, FADD, and PPFIA1 in invasive ductal carcinoma of the breast. To see the clinical implications of these gene expressions, we analyzed the relationship between the expressions and clinical parameters, including disease-free survival. As a result, we found that FADD expression was associated with T stage, showed a combined score of TMEM16A, FADD, and PPFIA1 expressions and was associated with perineural invasion and disease-free survival in the invasive ductal carcinoma cases.

According to the previous reports, high expression of FADD was associated with factors indicating poor prognosis in squamous cell carcinoma of the head and neck [14,27] and oral squamous cell carcinoma [28]. However, contradictory clinical associations of FADD expression were also reported. Colorectal cancer cell growth was inhibited by FADD expression [29] and high FADD expression after neoadjuvant breast cancer treatment was associated with a good prognosis group in breast cancer [30]. This may be due to the difference in cancer type or primary site from which the tumor has arisen. We found that FADD-positive cases showed a tendency towards better prognosis, which is concordant with the claim that high FADD expression is associated with better prognosis in breast cancer treatment. This also justifies subtraction of FADD score from the sum of TMEM16A and PPFIA1 scores when we calculate the hazard score of the three gene expressions in this study. Because the hazard score was associated with perineural invasion status of invasive ductal cancer of the breast, we may speculate that combination of TMEM16A, FADD and PPFIA1 expressions influence the invasiveness of the tumor cells and affect the ultimate prognosis of these cases. *In vitro* study at molecular level is required to confirm this possibility.

PPFIA1 is required for the migration and invasion of breast cancer cell lines [23]. However, as far as we know, prognostic significance of PPFIA1 expression in breast cancer has not been evaluated to date. PPFIA1 amplification was only reported to have significant association with poor prognosis in ER-positive cancer and PPFIA1 expression in terms of prognosis was not studied [31]. In this study, we showed that PPFIA1 expression showed a tendency towards an association with poor prognosis.

In addition to PPFIA1, significance of TMEM16A expression in breast cancer has not been studied to date. Here, we showed that TMEM16A expression also showed a tendency towards an association with poor prognosis as with PPFIA1. Although the individual gene expressions failed to show statistical significance of association, the combined effect of TMEM16A, FADD, and PPFIA1 gene expression was significantly associated with disease-free survival.

The regulatory mechanism of gene expressions between *PPFIA1*, *FADD*, and *TMEM16A* is not known. We cannot explain causal relationship between *PPFIA1*, *FADD*, and *TMEM16A* with our results in this study. However, we showed that combined expression status was associated with the disease-free survival in breast cancer. An explanation for this may be that the three genes located in the same amplification locus interact each other and influence tumor cell behavior in breast cancer; this effect resulting in the difference in disease-free survival.

An evaluation strategy of combining two or more gene alterations to analyze clinical significance can be found in several studies on human malignancies. Studies on the combination effects of two or more gene amplifications in 11q13 were conducted in oral squamous cell carcinoma [32] and breast cancer [31]. This method takes into account possible unknown interactions of other genes and strengthens statistical power in relatively small study sizes. Because every single gene expression is more or less affected by other gene expressions, studies on combination effects of gene expressions may be closer to biological phenomenon occurring *in vivo* than studies on single gene effects.

## Conclusion

In summary, we screened *TMEM16A*, *FADD*, and *PPFIA1* expression in breast cancer and found that combination of the three gene expressions was associated with disease-free survival in invasive ductal carcinoma of the breast.

## Abbreviations

LVI: lymphatic vessel invasion; PNI: perineural invasion; ER: estrogen receptor; PR: progesterone receptor.

## Competing interests

The authors declare that we have no competing interests, financial or otherwise.

## Authors’ contributions

EJC performed the immunohistochemical staining, data collection and drafted the manuscript. JAY and SJ participated in the immunohistochemical staining. EKJ and HSW collected clinicopathological data. YHK collected the cases of invasive ductal carcinoma. SYK designed the study, analyzed the data and finalized the manuscript.
